# Increased separase activity and occurrence of centrosome aberrations concur with transformation of MDS

**DOI:** 10.1371/journal.pone.0191734

**Published:** 2018-01-25

**Authors:** Sabrina Ruppenthal, Helga Kleiner, Florian Nolte, Alice Fabarius, Wolf-Karsten Hofmann, Daniel Nowak, Wolfgang Seifarth

**Affiliations:** Department of Hematology and Oncology, University Hospital Mannheim, Heidelberg University, Mannheim, Germany; European Institute of Oncology, ITALY

## Abstract

*ESPL1*/separase, a cysteine endopeptidase, is a key player in centrosome duplication and mitotic sister chromatid separation. Aberrant expression and/or altered separase proteolytic activity are associated with centrosome amplification, aneuploidy, tumorigenesis and disease progression. Since centrosome alterations are a common and early detectable feature in patients with myelodysplastic syndrome (MDS) and cytogenetic aberrations play an important role in disease risk stratification, we examined separase activity on single cell level in 67 bone marrow samples obtained from patients with MDS, secondary acute myeloid leukemia (sAML), *de novo* acute myeloid leukemia (AML) and healthy controls by a flow cytometric separase activity assay. The separase activity distribution (SAD) value, a calculated measure for the occurrence of cells with prominent separase activity within the analyzed sample, was tested for correlation with the centrosome, karyotype and gene mutation status. We found higher SAD values in bone marrow cells of sAML patients than in corresponding cells of MDS patients. This concurred with an increased incidence of aberrant centrosome phenotypes in sAML vs. MDS samples. No correlation was found between SAD values and the karyotype/gene mutation status. During follow-up of four MDS patients we observed increasing SAD values after transformation to sAML, in two patients SAD values decreased during azacitidine therapy. Cell culture experiments employing MDS-L cells as an *in vitro* model of MDS revealed that treatment with rigosertib, a PLK1 inhibitor and therapeutic drug known to induce G2/M arrest, results in decreased SAD values. In conclusion, the appearance of cells with unusual high separase activity levels, as indicated by increased SAD values, concurs with the transformation of MDS to sAML and may reflect separase dysregulation potentially contributing to clonal evolution during MDS progression. Separase activity measurement may therefore be useful as a novel additional molecular marker for disease monitoring.

## Introduction

Myelodysplastic syndromes (MDS) comprise a heterogeneous group of malignant oligo-clonal hematopoietic stem cell (HSC) disorders characterized by impaired growth and differentiation of hematopoietic progenitors associated with peripheral blood cytopenias and an increased risk of transformation to acute myeloid leukemia (AML) [1–3]. The clinical heterogeneity is underlined by a complex genetic make-up involving more than 50 recurrently somatically acquired mutations that can occur in diverse combinations and recurrently affect genes involved in various cellular processes [1]. Recent studies have demonstrated that MDS arise from a small population of disease-initiating HSC with initiating mutations that lead to the development of clonal hematopoiesis. Such initiating events are followed by accumulation of additional cooperating mutations including cytogenetic lesions and eventual progression to an overt clinical disease [1, 4, 5]. Analyses of the mutational hierarchies at multiple time points of MDS evolution by whole exome and targeted deep-sequencing revealed a highly dynamic and therapy-responsive shaping of complex oligoclonal architectures [6, 7]. Despite initial clinical response to treatment with Lenalidomide and other drugs, patient’s bone marrow persistently remained clonal with rapid outgrowth of founder-, sub-, or even fully independent clones, indicating a therapy-related increased dynamic rate of clonal turnover. Thus, the transformation process from MDS to secondary acute myeloid leukemia (sAML) can be described as “clonal evolution in an expanding population”, a post-Darwinian principle, widely accepted for the majority of cancers [8, 9]. Although the mutational landscape in MDS can be considered as predictive variables in MDS progression, a strong prognostic marker with feasibility in clinical routine diagnostics facilitating regular molecular monitoring to guide treatment decisions in MDS is still needed [7]. To date, the revised International Prognostic Scoring System (IPSS-R) is the most widely used scoring system for cytopenia-related mortality and transformation to sAML. Taking into account the degree of cytopenia, the proportion of bone marrow blasts and the karyotype, it does not include information about somatic mutations in individual genes or other cellular aberrations that may be of predictive value [5, 7, 10, 11].

*ESPL1*/separase, a cysteine endopeptidase, is a key player of chromosomal segregation and centrosome duplication as described in detail previously [12–14]. In mitotic anaphase, it accomplishes proteolytic cleavage of protein Rad21 (radiation-sensitive mutant 21), a “glue” multi-protein complex that is responsible for cohesion of sister-chromatids and of mother and daughter centrioles [15–18]. Proper temporal and spatial activation of separase proteolytic activity warrants chromosomal fidelity and proper semiconservative centriole duplication [19]. Failure to do so results in premature segregation of chromatids and/or formation of anaphase bridges from lagging chromosomes [20]. Moreover, cell cycle uncoupled activation of separase can lead to aberrant centrosome numbers [21]. Both defects cause the emergence of aberrant karyotypes, a hallmark of most human advanced malignancies [22–24]. In human cancer, *ESPL1*/separase is frequently overexpressed and/or overactive resulting in deregulated proteolytic activity associated with supernumerary centrosomes, chromosomal missegregation and aneuploidy [20, 23, 25, 26]. Separase has been identified as an aneuploidy promoter that functions as an oncogene when overexpressed and hyperactive and renders cells susceptible for loss of key tumor suppressor gene loci associated with tumorigenesis and disease progression [27–29]. Furthermore, in a subset of patients with chronic myeloid leukemia (CML), enhanced proteolytic activity of separase has been found to correlate with clonal evolution and accelerated transformation from chronic phase (hyperplasia) to blast crisis suggesting a role as driver for leukemia progression [30].

In this study, we investigated the context between separase activity and MDS progression by comparatively analyzing separase proteolytic activity, karyotype, centrosomal, mutational and clinical status in a total of 67 bone marrow samples derived from MDS, sAML, *de novo* AML patients and corresponding healthy control donors. We further investigated whether measurement of separase proteolytic activity can be a novel biomarker for disease monitoring.

## Materials and methods

### Patients characteristics

Overall, 60 bone marrow samples derived from 54 patients were analyzed (Table 1). Of these, 37 patients were diagnosed with MDS (mean age 69 y, range 28–88 y, 65% male), 8 with sAML after previous MDS (mean age 70 y, range 45–80 y, 50% male) and 9 with *de novo* AML (mean age 57 y, range 25–82 y, 44% male). In addition, bone marrow cells of 7 healthy donors (mean age 65 y, range 24–88 y, 14% male) served as controls. All MDS patients (n = 37) were untreated and samples were collected at time of initial diagnosis. From 6 MDS patients (#1, #15, #24, #36, #38, #43) samples were available at two time points, i.e. initial diagnosis and during follow-up (compare Table 1). Of these, patients #15 and #36 were untreated at time of initial diagnosis and received azacitidine (Vidaza, 75 mg/m^2^ subcutaneous, daily for 7 days) after 12.5 and 11.4 months of follow-up, respectively [31]. Cytogenetic and mutational data were available from 98% (53/54) of the patients. Centrosomes, gene mutations and karyotype of patients were analyzed at initial diagnosis and during follow-up. 27 MDS patients (73%) had a normal karyotype, 10 (27%) showed an aberrant karyotype already at time of initial diagnosis. Two patients (#24 and #43) underwent karyotype evolution during transformation from MDS to sAML (after 1.1 and 7.8 months of follow-up). In the sAML and AML groups, 3/8 (37%) and 4/9 (44%) displayed aberrant karyotypes, respectively. Bone marrow samples were obtained with written informed consent in accordance with the declaration of Helsinki and appropriate Ethics Committee (Medizinische Ethikkommision II der Medizinischen Fakultät Mannheim der Ruprecht Karls-Universität Heidelberg, #2013-509N-MA from 2013-02-21) approvals from patients with MDS, AML and sAML. Density gradient centrifugation using Ficoll-Paque was performed to separate mononuclear cells from bone marrow specimen. CD34^+^ progenitor cells were isolated using the CD34 MicroBead Kit according to the user´s manual (Miltenyi Biotec, Auburn, CA, USA).

**Table 1:** Characterization of patient samples and healthy controls.

Ptsno	Sex	Age	Subtype	Karyotype / follow-up sample comment	Gene mutations (Bejar-Panel)	SAD (follow up)	CA [%]	Therapy at ID	Therapy follow-up
**MDS patients with normal karyotype (n = 27)**									
1	M	68	RAEB-I	46,XY[20]	SRSF2, ASXL1	14.8	12.1	none	
1*	M	68	sAML	46,XY[20], follow-up sample drawn 3.2 months post ID	SRSF2, ASXL1	16.3	n.d.		none
2	M	75	RCMD	46,XY[20]	SRSF2, TET2	8.2	25	none	
3	F	72	RARS-T	46,XX[20]	JAK2, SF3B1	6.6	3.7	none	
4	F	70	RA/RCMD	46,XX[20]	SF3B1	9.7	19.8	none	
5	F	58	RAEB-I	46,XX[20]	SF3B1, TP53	12.9	7.9	none	
6	F	80	RARS-T	46,XX[22]	SF3B1, TET2, JAK2	8.9	3.5	none	
7	M	66	RARS	46,XY[21]	SF3B1	9.1	6.8	none	
8	M	75	RCMD	46,XY[20]	SF3B1, TET2	9.9	7.8	none	
9	F	73	Early MDS	46,XX[20]	SRSF2, TET2	9.8	7.2	none	
10	M	63	RCMD	46,XY[20]	TET2	14.2	n.d	none	
11	F	58	RCMD/RARS	46,XX[23]	JAK2	5.1	n.d	none	
12	M	63	RCMD	46,XY[20]	neg	14.0	n.d	none	
13	M	80	RCMD/RARS	46,XY[20]	SF3B1, ASXL1	8.5	n.d	none	
14	M	64	RCMD	46,XY[20]	SRSF2, ASXL1, RUNX1	10.6	n.d	none	
15	M	67	RAEB-I/II	46,XY[20]	neg	11.9	n.d	none	
15*	M	67	sAML	46,XY[20], follow-up sample drawn 12.5 months post ID	neg	6.9	n.d.		Vidaza
16	M	28	RCMD	46,XY[20]	U2AF1	14.8	n.d	none	
17	M	76	RCMD	46,XY[20]	n.a.	9.5	n.d	none	
18	F	44	MDS-U	46,XX[20]	SF3B1, RUNX1	8.2	n.d	none	
19	M	85	RCMD	46,XY[20]	NRAS	6.8	n.d	none	
20	M	77	RCMD	46,XY[20]	neg	6.3	n.d	none	
21	M	66	RCMD	46,XY[20]	DNMT3A	14.6	n.d	none	
22	M	63	RAEB-I	46,XY[20]	SF3B1	5.7	n.d	none	
23	F	88	RCMD	46,XX[20]	ASXL-1, RUNX1	9.9	n.d	none	
24	F	79	RCMD	46,XX[21]	neg	10.3	n.d.	none	
24*	F	79	sAML	46,XX [18] 45,XX,del(5)(q14q34),r(6)(p?23?16),+der(6;21)(6pter->6q26::19?p13->19?p13::21p12->21qter),r(7)(p?15q?22),der(9;11)(q10;q10),t(13;19;21)(q14;?p13;p13),-16,del(17)(p12p13)[6]/44,XX,del(5)(q14q34),r(6)(p?23?16),+der(6;21)(6pter->6q26::19?p13->19?p13::21p12->21qter),r(7)(p?15q?22),der(9;18)(q10;p10), der(12;22)(q10;q10), t(13;19;21)(q14;?p13),-16,del(17)(p12p13)[4], follow-up sample drawn 1.1 month post ID	DNMT3A, TP53n.d.	14.6	n.d		none
25	F	75	RARS	46,XX[20]	SF3B1, KMT2A	9.3	6.8	none	
26	F	67	RCMD	46,XX[20]	ASXL1, RUNX1, STAG2, IDH2	6.0	3	none	
27	F	67	RCMD	46,XX[20]	ASXL1	16.7	n.d	none	
**MDS patients with aberrant karyotype (n = 10)**									
28	M	78	RARS-T	47,XY,+8[15]/46,XY[5]	SF3B1, DNMT3A	7.7	n.d	none	
29	F	83	RARS-T	46,XX,der(6)t(3;6)(q21;q27)[2]/46,XX[18]	SF3B1	14	7.1	none	
30	M	77	RCMD	46,XY,t(2;2)(p23;q32)[10]/47,XY,t(2;2)(p23;q32),+8[2]/46,XY[9]	SRSF2, ASXL1, RUNX1	7.8	4.6	none	
31	M	74	RAEB-II	47,XY,+8[2]/46,XY[19]	ASXL1	11.5	n.d	none	
32	M	68	RCMD	46,XY,del(13)(q14q32)[2]/46,XY[18]	U2AF1	16.3	8.4	none	
33	M	55	RAEB-II	47,XY,+19[10]/48,XY,+8,+19[8]/46,XY[2]	SRSF2	6.3	n.d	none	
34	M	52	RARS-T	46,XY,del(5)(q14),der(11)t(11;16)(q22;q12)[16]/46,XY[4]	SF3B1	7.5	n.d	none	
35	M	68	RCMD	45,X,-Y[8]/45,X,-Y,del(1)(p34p36)[12]	ASXL1, U2AF1	9	6.5	none	
36	M	82	RAEB-I/II	42,XY,der(1)inv(1)(p36q32)t(1;5)(p36p15),add(2)(q37),-10,-11,del(13)(q14),add(17q),add(18q),+mar[22]	neg	9.8	4.7	none	
36*	M	82	RAEB-I/II	42,XY,der(1)inv(1)(p36q32)t(1;5)(p36p15),add(2)(q37),-10,-11,del(13)(q14),add(17q),add(18q),+mar[22], follow-up sample drawn 11.4 months post ID	neg	7.7	n.d.		Vidaza
37	M	71	MDS-U	47,XY,+der(1)del(1)(p12p31)t(1;6)(p36;p12),der(1)del(1)(p12p36)t(1;6)(p36p12)del(6)(p12p25)[1]/47,idem,del(5)(q23q34)[13]/47,XY,+der(1)del(1)(p12p31)t(1;6)(p36;p12),der(1)del(1)(p12p36)t(1;6)(p36p12)der(5)t(5;7)(p13;q36),del(6)(p12p25),der(7)t(5;7)(p14;q31)[3]/46,XY[4]	neg	8.9	n.d	n.a.	
**sAML patients with normal/aberrant karyotype (n = 8)**									
38	M	64	MDS	46,XY[20]	ASXL1, SRSF2, RUNX1	10.6	12.4	none	
38*	M	64	sAML	46,XY[20], follow-up sample drawn 2.8 months post ID	ASXL1, SRSF2, RUNX1, IDH2	11.9	n.d.		none
39	M	80	sAML	46,XY[20]	neg	15.5	10.5	Vidaza	
40	F	79	sAML	47,XX,+11[13]/46,XX[8]	neg	9.3	11.8	HU, Decitabine	
41	M	74	sAML	46,XY[20]	NPM1, SRSF2, IDH2	14	n.d	none	
42	M	69	sAML	46,XY[20]	ASXL1, SRSF2	16.3	12.1	none	
43	F	45	MDS	46,XX[20]	SF3B1, RUNX1	8.2	n.d.	none	
43*	F	45	sAML	46,XX,t(2;3)(p16;q26),del(5)(q21q34), follow-up sample drawn 7.8 months post ID	SF3B1, RUNX1	11.6	n.d		none
44	F	73	sAML	46,XX[20]	neg	17.9	n.d	Decitabine	
45	F	79	sAML	45,XX,del(5)(q14q34),r(6)(p?23?16),+der(6;21), r(7)(p?15q?22),der(9;11)(q10;q10),t(13;19;21)(q14;?p13;p13),-16,del(17)(p12p13)[6]/44,XX,del(5)(q14q34),r(6)(p?23?16),+der(6;21),r(7)(p?15q?22),der(9;18)(q10;p10)der(12;22)(q10;q10),t(13;19;21)(q14;?p13),-16,del(17)(p12p13)[4]/46,XX[18]	TP53, DNMT3A	14.6	n.d	none	
**AML patients with normal/aberrant karyotype (n = 9)**									
46	F	53	AML	46,XX[25]	FLT3	7.6	n.d	none	
47	M	25	AML	46,XY[20]	FLT3	8.6	4.8	none	
48	M	69	AML	47,XY,+21[6]/46,XY[14]	RUNX1	6.5	n.d	none	
49	F	57	AML	46,XX[20]	NPM1	10.8	n.d	none	
50	M	66	AML	45,X,-Y,t(8;21)(q22;q22)[15]/46,XY[5]	neg	11.3	14.9	none	
51	M	39	AML	46,XY[35]	NPM1	9.1	n.d	none	
52	F	70	AML	46,XX[20]	neg	4.2	n.d	none	
53	F	51	AML	45,X,-X, t(8;21)(q22;q22)[17]/46,X,-X, t(8;21)(q22;q22)[6]	NPM1, FLT3	8.8	n.d	none	
54	F	82	AML	47,XX,+8[14]/46,XX[6]	ASXL1	7.3	n.d	none	
**Healthy donors (controls, n = 7)**									
55	F	76	healthy	n.d	neg	11.7	4.2	none	
56	M	85	healthy	45,X,-Y[9]/46,XY[11]	neg	13.1	5	none	
57	F	82	healthy	n.d	neg	11.1	4.9	none	
58	F	85	healthy	46,XX[15]	neg	10.7	5.1	none	
59	F	24	healthy	46,XX[20]	n.d.	10.9	5	none	
60	F	79	healthy	46,XX[20]	DNMT3A	11.5	4.9	none	
61	F	25	healthy	n.d	neg	11.8	n.d	none	

Abbreviations: Pts no, patients number; F, female; M, male; n.d, not done; n.a, not available; neg, no mutations detectable; ID, initial diagnosis; CA, centrosomal aberrations; PC, platelet concentrates; RBC, red blood cells; HU, Hydroxyurea; ARAC, Cytarabine

* indicates patient with follow-up bone marrow sample; MDS-U, myelodysplastic syndrome unclassified; sAML, secondary acute myeloid leukemia; RA, refractory anemia; RARS, refractory anemia with ring sideroblasts; RAEB, refractory anemia with excess of blasts; RCMD, refractory cytopenia with multilineage dysplasia.

### Cell lines and drug treatment

The human cell line MDS-L (derived from MDS92) that served as an *in vitro* model of MDS was obtained from Dr. Kaoru Tohyama (Japan). MDS-L cells are positive for CD34, c-Kit, HLA-DR, CD13, CD33 and partially positive for CD41 and negative for CD3, CD14, CD20 and glycophorinA. The main karyotype was aberrant with 49, XY, +1, der(5)t(5;19), −7, +8, −12, der(13)t(7;13), der(14)t(12;14), der(15)t(15;15), +19, +20, +21, der(22)t(11;22). Multicolor fluorescence in situ hybridization analysis indicated that MDS-L does not show a simple deletion of the single 5q locus but reveals a derivative small chromosome 5 as a result of t(5;19)(q11;q13). Fluorescence in situ hybridization analysis targeting the 5q locus also indicated that the distal portion from 5q11.1 was certainly lost [32, 33]. Cells were cultured in complete RPMI-1640 medium (Gibco/ThermoFischer Scientific), supplemented with 10% fetal bovine serum, 1% penicillin-streptomycin (Gibco/ThermoFischer Scientific) and 50 ng/ml GM-CSF (Gibco/ThermoFischer Scientific), at 37°C in 5% CO_2_ atmosphere. For optimal proliferation cells were maintained at a density of about 3x10^5^ cells/ml. For drug treatment experiments 5x10^5^ cells/ml were propagated in the described culture medium containing one of the therapeutic drugs (Selleckchem.com/Absource Diagnostics GmbH, Munich, Germany) azacitidine, lenalidomide or Rigosertib at concentrations of 500 nM for 48 h. Untreated cells served as controls. Numerous pilot experiments have been performed for dose and incubation time optimization (data not shown) in analogy to reports of others [33, 34]. Due to the antiproliferative and apoptotic effects of the drugs we had to select drug concentrations and incubation times that granted enough vital cells for conducting the flow cytometry experiments for separase activity measurements. 11% of MDS-L cells display an aberrant centrosomal phenotype.

### Apoptosis assay

The apoptosis was examined using AnnexinV Apoptosis Detection Kit (BD Pharmingen, San Diego, CA, USA). All samples were analyzed by FACS Calibur flow cytometer and Kaluza software (Version 1.3, Beckman Coulter, Inc., Krefeld, Germany).

### Cell cycle analysis

Subconfluent MDS-L cells were harvested and washed in 1x phosphate buffered saline (PBS), subsequently fixed in icecold 75% ethanol and stained with propidium iodide (10 μg/ml propidium iodide, 2 mg/ml RNAse A in PBS). DNA content was measured by fluorescence-activated cell sorting (FACS) using a flow cytometer FACSCalibur (Becton Dickinson, San José, USA). Cell cycle analysis was performed with Flowing Software version 2.5.1 (by Perttu Terho, Turku, Finland).

### Measurement of separase proteolytic activity

The flow cytometric separase activity assay was performed according to our standardized protocol as previously described [35]. In brief, 2x10^5^ mononuclear bone marrow cells were resuspended in 200 μl of complete RPMI-1640 medium containing a protease inhibitor cocktail (PIC) selective for serine-/threonine proteases (Pefabloc SC, #76307 Sigma), Trypsine-/Chymotrypsine (#T9777, Sigma, Taufkirchen, Germany) and MMP 2/MMP 9 matrix metalloproteases (MMP-2/MMP-9 Inhibitor III, #444251 Calbiochem/Merck, Darmstadt, Germany). The final concentrations were 250 μM, 25 μM and 20 μM, respectively. After 5 min of PIC pre-incubation the fluorogenic peptide was added and cells were incubated for 90 min at 37°C in 5% CO_2_ atmosphere. Fluorescence (20.000 events/sample) of Rh110 (Ex_max_ 488 nm, Em_max_ 535 nm) was measured by flow cytometry (FACSCalibur, Becton Dickinson, San José, USA). Data analysis was performed by Kaluza software (Version 1.3, Beckman Coulter, Inc., Krefeld, Germany). FSC files were imported in Microsoft Excel 2010 (Microsoft, Redmond, WA, USA) for further analysis [35]. After subtraction of dead/apoptotic cell fractions and the baseline signals of the cellular autofluorescence gating controls, the separase activity distribution (SAD) value of the remaining vital cells was calculated as quotient of the mean relative fluorescence units (RFU) of 0.5% separase positive cells above the 99.5 percentile divided by mean (M) RFU of 99.5% of separase positive cells below the 99.5 percentile (SAD value = M_0.5_/M_99.5_). Thus, the SAD value serves as a numerical measure of intercellular separase activity distribution among single cells in the sample of interest.

### Centrosome staining

CD34^+^ interphase cells were centrifuged on PTFE-coated slides (ThermoFisher Scientific, Waltham USA), fixed and permeabilized. Immunofluorescence was performed as previously described using primary antibodies to pericentrin (Abcam ab4448, Cambridge, UK) and alpha tubulin (Sigma T6074, Taufkirchen, Germany) followed by Alexa fluorophore-conjugated secondary antibodies (Alexa Fluor®, ThermoFisher scientific, USA) [36]. At least 100 cells per sample were examined by fluorescence microscopy (JenovalOpton ID02, Zeiss, Oberkochen, Germany). Centrosomal phenotypes were considered abnormal, if centrosome-related signals were present in numbers >2. Centrosomal alteration were also found in up to 5% of analyzed cells of the healthy controls and were therefore evaluated as normal (5% = cut-off).

### Cytogenetics

Cytogenetic analyses of 20–25 G-banded bone marrow metaphases (24- and/or 48 h culture) were interpreted according to the International System for Human Cytogenetic Nomenclature [37]. In case of complex aberrant karyotypes chromosome banding analysis was combined with fluorescence in situ hybridization (FISH) analysis according to the manufacturer´s instructions (Metasystems, Altlussheim, Germany).

### Gene mutation status of patient bone marrow samples

All data on the gene mutations status of the patients in this study was kindly provided by the MLL Munich Leukemia Laboratory GmbH (Munich, Germany) where bone marrow and/or peripheral blood samples were routinely sent for diagnosis. Gene mutation analyses were performed by next-generation amplicon deep-sequencing (Illumina, San Diego, CA, USA; 454 Life Sciences, Branford, CT, USA; sensitivity: 3%) applied for a myeloid gene panel comprising the genes *ASXL1*, *BCOR*, *CALR*, *CBL*, *CEBPA*, *DNMT3A*, *ETV6*, *EZH2*, *FLT3*-ITD, *FLT3*-TKD, *GATA2*, *IDH1*, *IDH2*, *JAK2*, *KIT*, *KRAS*, *MLL*-PTD, *MPL*, *NPM1*, *NRAS*, *RUNX1*, *SETBP1*, *SF3B1*, *SRSF2*, *STAG2*, *TET2*, *TP53*, *U2AF1*, *WT1* and *ZRSR2* as described previously [38].

### Statistical analysis

Statistical calculations were done with GraphPad Prism version 6.0 (GraphPad Inc., La Jolla, USA) or SAS software, release 9.3 (SAS Institute Inc., Cary, NC, USA). Quantitative parameters are presented as mean values together with standard deviation (SD) or 95% confidence intervals (CI). A two-tailed unpaired t-test was used to compare SAD values and centrosomal aberrations between different groups. P values of less than 0.05 were considered statistically significant. The correlation coefficient (r) was calculated using Pearson’s correlation analysis. In order to evaluate the differences in the centrosome status between healthy, MDS and sAML groups we performed the Kruskal-Wallis test. Mann-Whitney U tests followed by Bonferroni-Holm p-value correction were made as post-hoc tests in order to compare the MDS and sAML patients with the control group.

## Results

### Elevated SAD values are found in sAML when compared to MDS, AML patients and healthy controls

In order to investigate the potential context between altered separase activity and MDS progression we have comparatively analyzed separase proteolytic activity, karyotype, centrosomal, mutational and clinical status in a total of 67 bone marrow samples derived from 54 patients with MDS, sAML, *de novo* AML and from 7 corresponding healthy control donors. For 6 MDS patients (#1, #15, #24, #36, #38, #43) one follow-up sample for each (n = 6) was available and analyzed (Table 1).

**Fig 1:**
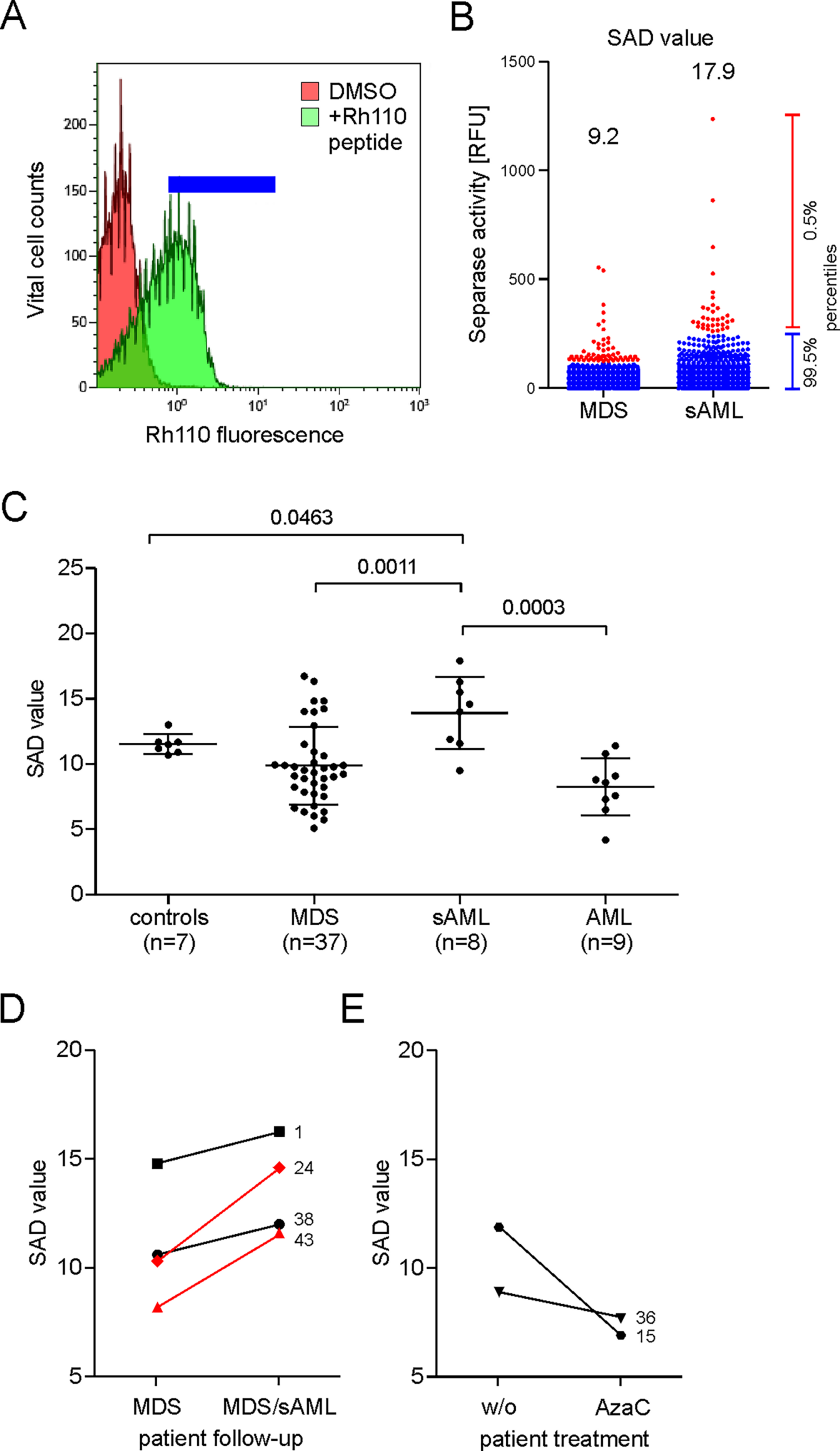
Analysis of separase activity distribution in vital separase-positive MNC fractions derived from bone marrow samples of patients with MDS, sAML, AML and of healthy donors. (A) Flow cytometric histogram of MNCs after incubation with a rhodamine 110 (Rh110)-conjugated separase-specific substrate (green), compared to untreated cells (DMSO control in red). Gating for separase-active cells is depicted by the blue horizontal bar. (B) Visualization of separase activity distribution on single cell level relating to flow cytometric data sets as exemplarily shown in (A) and SAD value calculation. The dot blots represent separase-active cells ordered by their Rh110 fluorescence. The distribution of Rh110 intensities has been accentuated by coloring cells above the 99.5 percentile (= upper 0.5% of Separase positive cells) in red and cells below the 99.5 percentile (= 95.5% of Separase- positive cells) in blue. The quotient of mean Rh110 fluorescence intensities (mean_0.5%_/mean_99.5%_) was calculated to serve as numerical value of cellular separase activity distribution in the clinical samples under investigation. Here, the sAML sample includes cells with higher intrinsic separase activity than the MDS sample resulting in calculation of a higher SAD value (17.9 vs. 9.2). (C) Comparative analysis of SAD values of clinical specimen (MDS, n = 7; sAML, n = 8; AML, n = 9) and healthy controls (n = 7). (D) SAD value comparison of clinical sample pairs (n = 4) each pair derived from the same patient (patient IDs #1, #24, #38, 43) but drawn at two different time points during clinical follow-up (MDS at initial diagnosis and after progression to sAML). Patients #24 and #43 (shown in red) underwent karyotype evolution during transformation from MDS to sAML. Numbers correspond to patient IDs shown in Table 1. (E) SAD value comparison of clinical sample pairs (n = 2) each pair derived from the same MDS patient (#15, #36) but drawn at two different time points (before (w/o) and under treatment with azaciditine (AzaC)). Numbers correspond to patient IDs as listed in Table 1. Abbreviations: RFU, relative fluorescence units; AzaC, azacitidine; SAD, separase activity distribution.

For assessment of separase proteolytic activity we have employed a flow cytometry-based assay that utilizes a highly specific rhodamine 110 (Rh110)-conjugated synthetic peptide as intracellular substrate for detection and relative quantification of Separase enzyme activity in single living cells. Using a standardized protocol the highly sensitive assay delivers equivalent results when compared to conventional cell extract-based methods but is more reliable, bypasses the problem of vague loading controls and unspecific proteolysis associated with whole cell extracts as demonstrated previously [35]. The FACS assay allows generation of Separase activity profiles that not only tell about the number of Separase positive cells within a sample but also about the range of intercellular variation in Separase activity levels within a cell population. The assay was used to quantify the Separase proteolytic activity in mononuclear cells (MNCs) of clinical specimen and to calculate the SAD value that serves as a numerical measure of intercellular separase activity distribution among single cells in the analyzed sample (Fig 1A and 1B). In other words, the SAD value is a calculative value for the occurrence of cells with prominent Separase activity even though the number of these cells may be low. As shown in Fig 1C measurement of separase activities and comparison of calculated SAD values between healthy controls (n = 7) and patients with MDS (n = 37), sAML (n = 8) and *de novo* AML (n = 9) revealed higher SAD values in sAML (mean 13.9 ± 0.99, range 9.3 to 17.9) samples when compared to the control group (mean 11.6 ± 0.29, range 10.7 to 13.0, p = 0.0463), the MDS group (mean 10.0 ± 0.49, range 5.1 to 16.7, p = 0.0011), and the *de novo* AML group (mean 8.2 ± 0.76, range 4.2 to 11.4, p = 0.0003) indicating an association between high SAD values and disease progression in MDS patients. The phenotype of *de novo* AML differs from that of sAML as the MNC samples of these patients do not show elevated SAD values.

### SAD values concur with disease severity in MDS follow-up samples

For six MDS patients follow-up samples were available for pairwise analysis. Of these, four clinical sample pairs, each pair derived from the same patient (patient IDs #1, #24, #38, #43) but drawn at two different time points (MDS vs. sAML) were tested (Fig 1D). The SAD values of all patients increased during course of the disease when compared to the values calculated from samples derived from time of diagnosis. MDS patients #24 and #43 underwent karyotype evolution during transformation to sAML (compare Table 1). For two patients (#15, #36) follow-up sample pairs were available corresponding to time points before and under treatment with azaciditine (Fig 1E). Azacitidine (Vidaza) treatment caused a drop in SAD values pointing to a drug-related reduction of MNC numbers with prominent Separase activity in the analyzed MDS samples.

### Increased occurrence of aberrant centrosome and karyotype phenotypes in sAML compared to MDS and healthy controls

**Fig 2:**
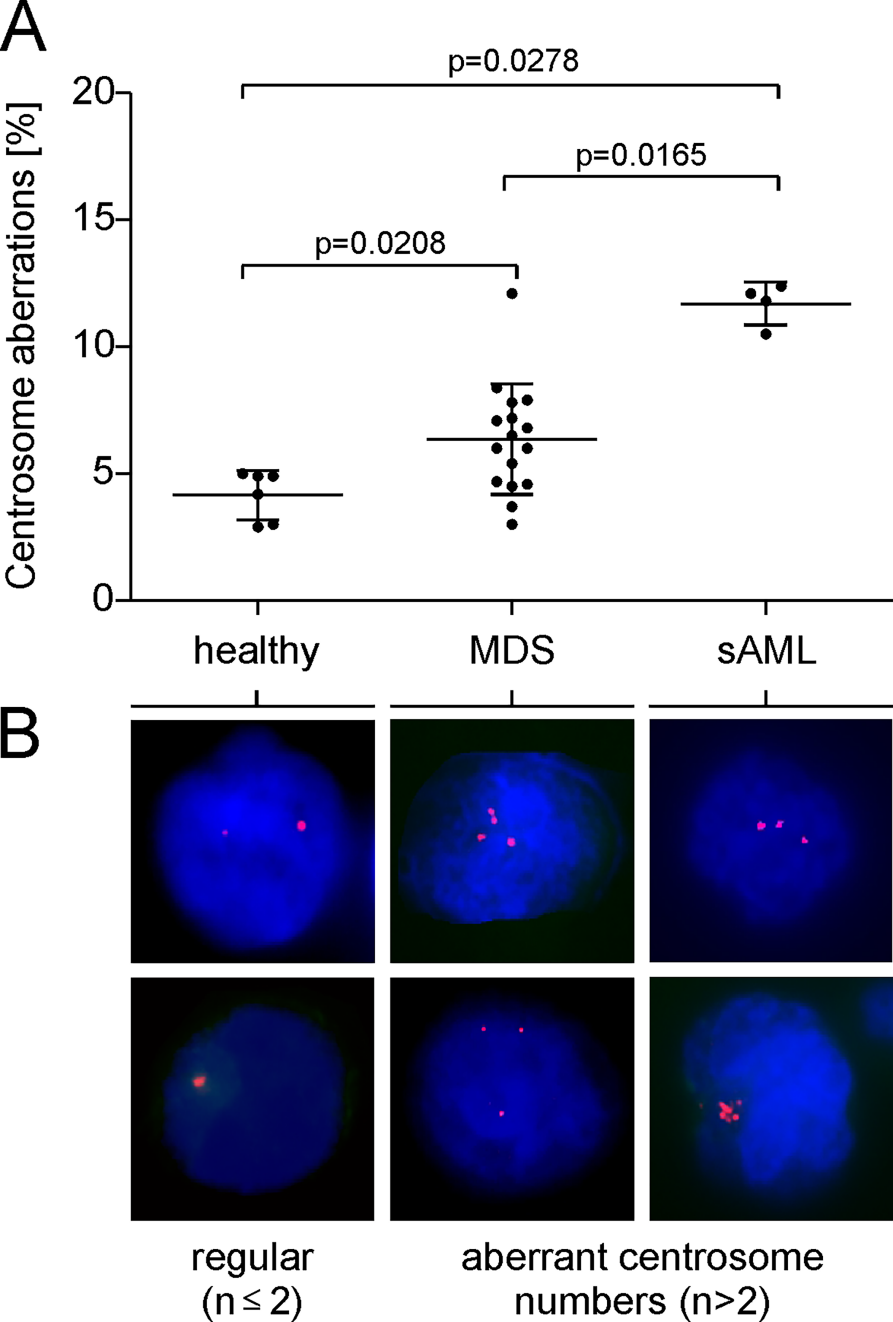
Occurrence of aberrant centrosomal phenotypes in CD34^+^ bone marrow cells. (A) Centrosome staining and indirect immunofluorescence microscopy was performed on normal bone marrow samples (healthy controls, n = 6) and on specimen derived from patients with MDS (n = 16) and sAML (n = 4). Centrosome alterations in ≤ 5% of the analyzed interphase cells (n = 100) were evaluated as normal. (B) A representative panel of indirect immunofluorescence microscopic images shows normal (regular, n ≤ 2) and aberrant centrosome numbers (n > 2) in interphase cells. Centrosomes were stained using anti-pericentrin antibody (magenta), nuclear DNA is shown in blue (DAPI). Statistical methods: Kruskal-Wallis test. Mann-Whitney U tests followed by Bonferroni-Holm p-value correction were made as post-hoc tests in order to compare the MDS and sAML patients with the control group.

In order to investigate the potential context between the centrosomal status, altered separase activity and MDS progression we have investigated the centrosomal phenotype of 26 clinical specimen including 16 MDS patients, 4 patients with sAML and 6 healthy controls. Interphase CD34+ cells derived from bone marrow samples were immunostained with a centrosome-specific antibody to pericentrin (Fig 2.). Due to clinical sample limitations, not for all bone marrow samples described in Fig 1C corresponding cells for pericentrin immunostaining experiments were available. Aberrant centrosomal phenotypes were detected in 6% of analyzed cells (n = 100) of MDS samples (range 3 to 13.1), in 12% of cells of sAML samples (range 10.5 to 12.4) but in only 4% (range 2.9 to 5.0) of analyzed cells of healthy controls. Since all MDS samples of this study (except the six follow-up samples #1, #15, #24, #36, #38, #43) are derived from the time point of diagnosis, our results confirm previous data indicating that centrosome alterations are a common and early detectable feature in MDS patients [39]. Aberrant karyotypes were found in 5 of 16 MDS samples (#29, #30, #32, #35, #36) and in 1 of the 4 sAML (#40) samples. Details are given in Table 1. When comparing the karyotype status of all patients (n = 54) investigated in this study aberrant karyotypes were found in 27% (10/37) of MDS patients, in 38% (3/8) of sAML patients and in 44% (4/9) of the patients with *de novo* AML.

For the majority of patients investigated in this study, clinical information about the gene mutations status was available featuring data on a myeloid gene panel comprising the genes *ASXL1*, *BCOR*, *CALR*, *CBL*, *CEBPA*, *DNMT3A*, *ETV6*, *EZH2*, *FLT3*-ITD, *FLT3*-TKD, *GATA2*, *IDH1*, *IDH2*, *JAK2*, *KIT*, *KRAS*, *MLL*-PTD, *MPL*, *NPM1*, *NRAS*, *RUNX1*, *SETBP1*, *SF3B1*, *SRSF2*, *STAG2*, *TET2*, *TP53*, *U2AF1*, *WT1* and *ZRSR2*. As recorded in detail in Table 1, gene mutations occurred in 86% (31/36) of MDS patients, in 63% (5/8) of sAML patients and in 100% (9/9) of patients with *de novo* AML. In 39% (14/36) of MDS patients, only one gene was mutated, two gene mutations were found in 36% (13/36) patients and 11% (4/36) of the MDS patients had three or more mutations.

### Occurrence of aberrant centrosome phenotypes and SAD values positively correlate in MDS patients

**Fig 3:**
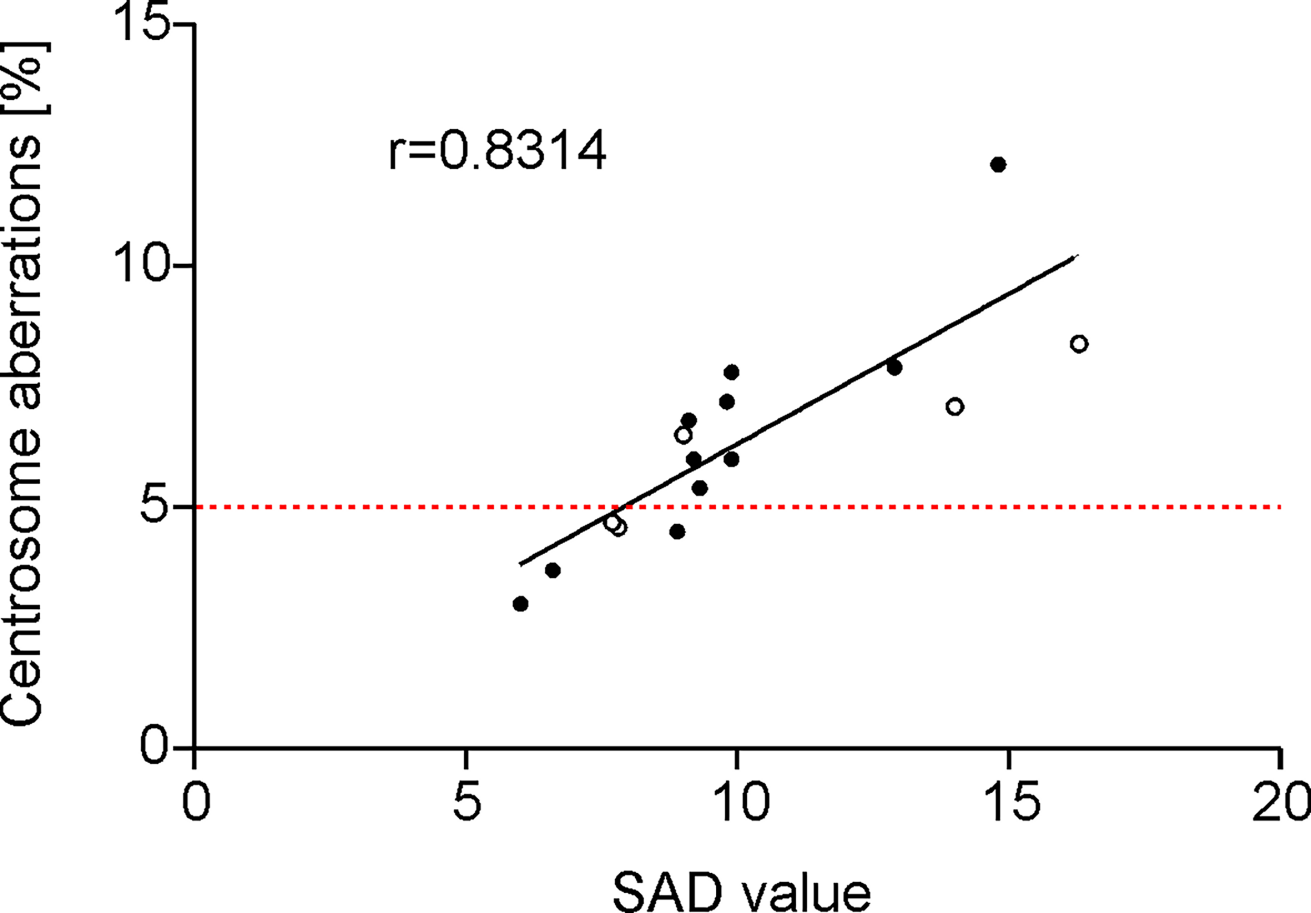
Correlation between separase proteolytic activity distribution (SAD value) and the occurrence of aberrant centrosomal phenotypes in patients with MDS (n = 16). Open circles indicate patients with karyotype aberrations (compare patient IDs #29, 30#, #32, #35, #36 as listed in Table 1). Method: Pearson correlation analysis, r = 0.8314 (CI95%: 0.7360 to 0.9269). The 5% cut-off value for specification of normal/aberrant centrosome phenotypes is depicted by the red dotted line.

For 16 MDS patients complete data sets including SAD value, centrosomal and karyotype status were available. Of these, 13 samples showed an aberrant centrosome phenotype (cut-off 5%), 5 samples (patients #29, 30#, #32, #35, #36 as listed in Table 1) had aberrant karyotypes (depicted by open circles in Fig 3). Pearson correlation analysis revealed a positive correlation between SAD value and the occurrence of an aberrant centrosome phenotype (r = 0.8314 (CI95%: 0.7360 to 0.9269)). Due to the limited sample size and high heterogeneity of the mutational data set, no correlation could be stated between SAD values and occurrence of karyotype aberrations/number and type of gene mutations.

### Drug treatment of MDS-L cells with rigosertib reduces the SAD value via G2/M arrest

**Fig 4:**
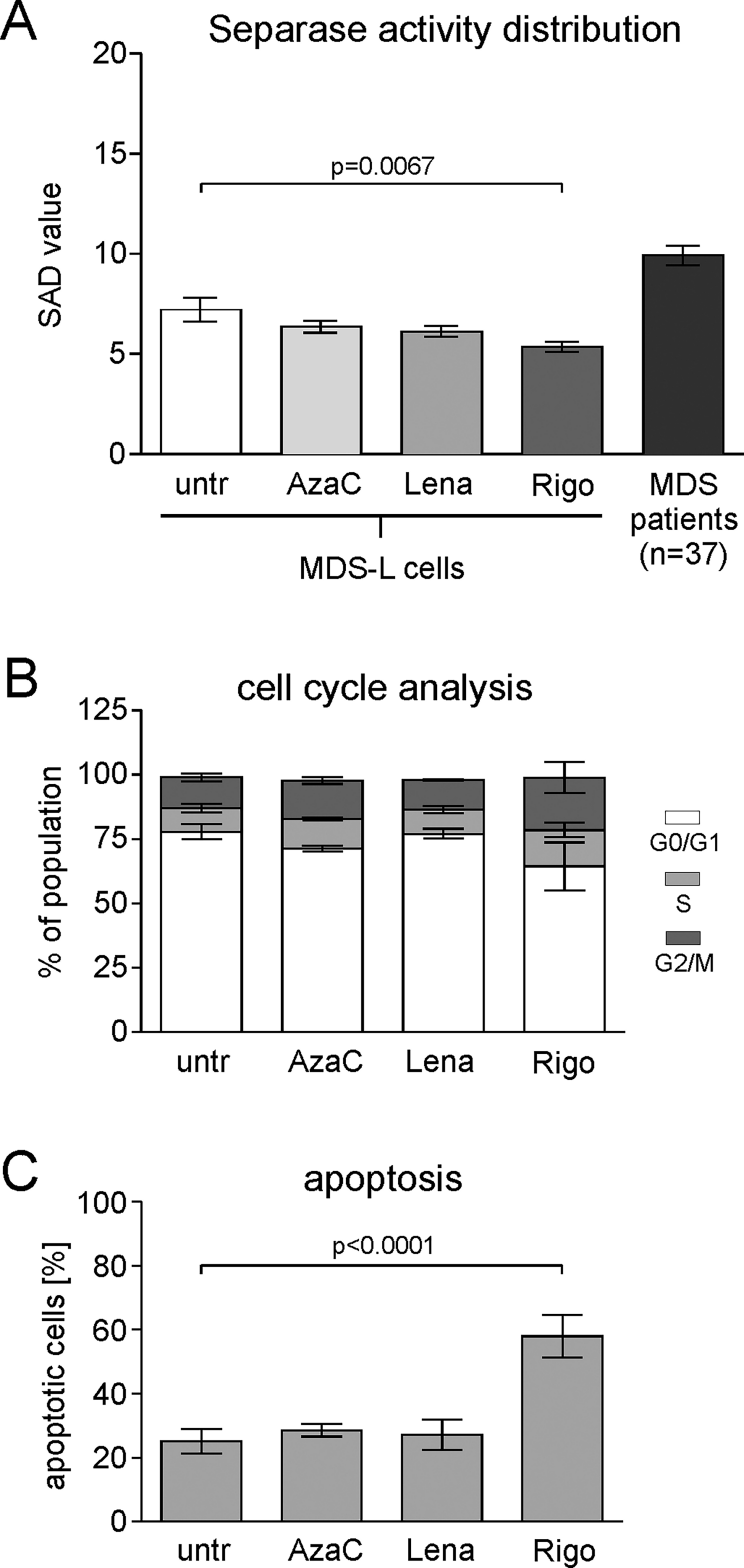
Influence of the therapeutic agents azacitidine, lenalidomide and rigosertib on the separase activity distribution (SAD value) in MDS-L cell culture experiments. (A) Exponentially growing MDS-L cells were treated with azacitidine, lenalidomide and rigosertib, each at concentrations of 500 nM for 48 hours. Subsequently, separase proteolytic activity was analyzed in the remaining vital cell fraction. Untreated MDS-L cells served as negative control (untr). As *in vivo* benchmark, the mean over the SAD values of all MDS patients under investigation (n = 37) is presented by the very right column. Method: Unpaired two-tailed t test, p = 0.0067 (CI95%: -3.159 to -0.5398). The data are derived from 11 independent assay experiments, each performed in duplicates. (B) The corresponding cell cycle profiles of treated MDS-L cells were analyzed by flow cytometry after propidium iodide (PI) staining. The cell fractions at G1, S and G2/M phase are represented by white, gray and black fillings, respectively. Data are derived from 2 independent assay experiments performed in duplicates. (C) Assessment of apoptosis in treated MDS-L cells by flow cytometry using annexinV / PI staining. Data from early (annexinV positivity only) and late apoptosis (double-positive fraction for annexinV and PI) were combined and are derived from 2 independent assay experiments performed in duplicates. Abbreviations: SAD, separase activity distribution; untr, untreated; AzaC, azacitidine; Lena, lenalidomide; Rigo, rigosertib.

In order to investigate whether the drugs azacitidine, lenalidomide and rigosertib, all commonly used in MDS treatment, are able to modulate the SAD value, i.e. the occurrence of bone marrow cells with prominent separase activity *in vivo*, we performed cell culture experiments on MDS-L cells, a human myeloid cell line best suitable as an *in vitro* model system of MDS [32]. Numerous pilot experiments have been performed for dose and incubation time optimization (data not shown) in analogy to reports of others [33, 34]. Due to the antiproliferative and apoptotic effects of the drugs we had to select drug concentrations and incubation times that granted enough vital cells (apoptotic rate ≤ 50%) for conducting the flow cytometry experiments for separase activity measurements. Exclusively viable cells were gated in the flow cytometry-based separase activity assay. After treatment for 48 h with 500 nM of the respective drug MDS-L cells were subjected to the standardized separase activity assay. In parallel, cell cycle status and proportion of apoptotic cells were analyzed by flow cytometry after propidium iodide (PI) and annexin V/PI staining, respectively. As shown in Fig 4A treatment with the PLK1 inhibitor rigosertib resulted in a drop of the SAD value (5.415 ± 0.2484, p = 0.0067) when compared to untreated MDS-L cells (7.265 ± 0.5991). No significant SAD value changes were observed after treatment with azacytidine (6.630 ± 0.2996) and lenalidomide (6.416 ± 0.3363).

Except for transformed tumor cells where unscheduled and cell cycle-independent activation of separase proteolytic activity is known to contribute to centrosome amplification, defective mitotic spindles and aneuploidy, in non-malignant cells separase is activated for a short period at anaphase onset just once per cell cycle [20]. Cell cycle analysis (Fig 4B) revealed that the decline of SAD values under treatment with rigosertib concurred with a G2/M arrest as the number of cells in G2/M phase nearly doubles (20.5% in G2/M) when compared to untreated MDS-L cells (12% in G2/M). A similar, but weaker effect was found after azacitidine (15% of cells in G2/M), but not after lenalidomide treatment (11.4% in G2/M). The observed cell cycle arrest can well explain reclined SAD values as fewer cells will undergo G2/M transition to enter mitosis.

Rather, cells may enter the apoptotic pathway as confirmed in Fig 4C where treatment of MDS-L cells concurred with considerable apoptosis (57.95 ± 3.325, p<0.0001) compared to corresponding untreated cell cultures (25.17 ± 1.899). No apoptotic effects were detected in azacitidine (28.60 ± 0.9873) and lenalidomide-treated (27.16 ± 2.346) MDS-L cell cultures. Drug treatment for 48 h did not influence the complex aberrant karyotype and the centrosomal status (11% of cells with aberrant centrosomal phenotype) of MDS-L cells.

## Discussion

Although cytogenetic aberrations and an increasing number of gene mutations play an important role in MDS risk stratification, there is still a need for additional molecular markers that may be useful to reliably predict progression of MDS to sAML for the individual patient. In search for novel predictive markers we have investigated the context between separase activity and MDS progression by comparatively analyzing separase proteolytic activity, karyotype, centrosomal, mutational and clinical status in a total of 67 bone marrow samples derived from MDS, sAML, *de novo* AML patients and corresponding healthy control donors.

For measurement of separase proteolytic activity, we employed a flow cytometry-based real-time assay for detection and quantification of separase enzyme activity in living cells. While previous cell extract-based separase assays were able to measure separase activity as an average of all cells lysed during whole cell extract preparation only, this single cell-based FACS assay allows identification of separase positive cell counts on the one hand and the monitoring of the range of intercellular variation in separase activity levels within the tested cell population on the other hand, i.e. the detection of even small numbers of cells with prominent levels of separase proteolytic activity [25, 35]. This technical advantage allowed us to design the present study to investigate separase proteolytic activity in vital MDS and sAML bone marrow cells and to calculate the separase activity distribution (SAD) value as a numerical measure of intercellular separase activity distribution among single cells in the analyzed sample. The resulting separase activity profiles gave information about the occurrence of cells with prominent Separase activity even though the number of these cells was low. Due to limitations in the available amounts of diagnostic material and the fact that fresh and unfrozen diagnostic material had to be used for unbiased separase activity testing results, not all types of analyses could be performed with each patient sample. In this context, it is to note that only a minor fraction of the analyzed bone marrow cells is mitotically active i.e. separase-positive and therefore, a high number of bone marrow cells had to be subjected to flow cytometric analysis according to our standardized protocol [35].

Since the specificity and reliability of the enzymatic separase activity assay is crucial and represents a potential weakness of this study, it should be emphasized that during assay establishment numerous experiments have been performed to exclude the possibility that further intra- or extracellular proteases will unspecifically cleave the fluorogenic substrate, thereby giving rise to false positive signals [35]. Although we cannot completely exclude the possibility that other intracellular proteases might potentially contribute somewhat to the Rh110 substrate cleavage as already acknowledged by assay descriptions of others [25], testing of various peptidic substrates and protease inhibitor combinations on synchronized cells with varying separase expression levels let us consider that the separase FACS assay according to our standard protocol is in fact specific and a functional tool for the quantitative analysis of separase activity levels in blood- and bone marrow-derived hematopoietic cells [35].

We found higher SAD values in the bone marrow cells of sAML patients than in the corresponding cells derived from MDS patients (p = 0.0011). This concurred with an increased incidence of aberrant centrosomal phenotypes in sAML compared to MDS samples (p = 0.0165, r = 0.8314). The increased SAD values in sAML samples indicate the existence of a small number of bone marrow cells with prominent levels of separase proteolytic activity when compared to levels regularly measured in corresponding cells of MDS patients and healthy controls. Those cells may either overexpress *ESPL1*/separase or may have defects in the cell cycle-dependent posttranslational regulation of separase. Overexpression or unscheduled separase activity in a small number of bone marrow cells may serve as driver of centrosomal aberration, chromosome missegregation, potentially contributing to tumor heterogeneity and clonal evolution [20, 23, 27]. These bone marrow cells may give rise to the emergence of aneuploid tumor cell progeny with enhanced fidelity to escape therapeutic pressure as suggested previously [39]. Interestingly, *de novo* AML samples displayed regular SAD values comparable to those found in MDS, healthy controls and patients after SCT (data not shown) underlining different pathomechanisms of *de novo* AML when compared to sAML.

Our finding that the transformation process of MDS to sAML is accompanied by enhanced SAD values was further corroborated by monitoring disease progression in six patients (follow-up cohort) who were initially diagnosed with MDS. Four developed a sAML in the course of the present study. As a matter of fact, in these patients we observed increased SAD values in all bone marrow samples at the time of progression (sAML) as compared to the time of initial diagnosis (MDS). These results suggest that the number of cells with prominent levels of separase proteolytic activity may rise during the transformation process from MDS to sAML. Since separase dysregulation has been reported to induce chromosomal instability [20] via defective centriole and sister chromatid separation during mitotic anaphase we hypothesize the occurrence of cells with enhanced separase activity as an additional driver of and a potential new marker for progression in MDS. Analogous observations have been reported for chronic myeloid leukemia (CML) where clonal evolution and progression time from chronic phase to blast crisis correlated with enhanced proteolytic activity of separase in patients with BCR-ABL e14a2 fusion type CML under treatment with the tyrosine kinase inhibitor imatinib [30].

However, we found no correlation between the incidence of gene mutations and the SAD value. Given the heterogeneity of the MDS/sAML-related mutational gene panel the small data set of this study does not allow to make a statement about the functional relationship between separase activity and the observed mutations. Nevertheless, for the individual patient, mutations in *ASXL1*, *RUNX1* or *SRSF2* can be associated with transformation and a shorter progression free survival [40].

Karyotype evolution is a common hallmark of the transformation process in pre-malignant neoplasia. However, karyotype aberrations could not be observed in all centrosome aberrant samples under investigation. In two patients of our follow-up cohort, karyotype evolution concurred with increased separase activity exemplifying that clonal evolution may concur at least in some cases together with high SAD values. Unfortunately, for both patients no complete data on the centrosome status was available making interpretation of causality in terms of a “step by step” mechanistic pathway impossible. The observation that centrosomal aberrations do not necessarily coincide with karyotype aberrations is in accordance with previous findings that the occurrence of supernumerary centrosomes is an early event and can be observed before chromosomal changes become detectable [39]. Keeping in mind that the majority of the analyzed MDS bone marrow samples have been derived from the time point of diagnosis, this may explain the observation that only 3 of 11 patients (27%) with aberrant centrosome phenotypes (compare Fig 3, open circle dots above 5% cut-off) feature an aberrant karyotype as well.

It is to be noted that assessment of centrosomal aberrations has been performed on CD34+ interphase cells by pericentrin staining, a protein of the pericentriolar matrix such as gamma-tubulin. Although this has been a commonly used approach for monitoring centrosome amplification with respect to disease association [36, 39, 41–44], it does not allow statements about the functionality and the pathomechanistic impact of the observed supernumerary centrosomes, i.e. causing multipolar catastrophic mitoses. Characterization of centrosome abnormalities in various cancer cell lines has revealed that supernumerary centrosomes, when devoid of centrioles, were unable to nucleate microtubules despite the presence of sufficient gamma-tubulin, pericentrin, PLK1 and AURKA proteins [45]. Therefore, without proof of the presence of centrioles and the capacity to nucleate microtubules, the observation of aberrant centrosome numbers must remain a mere phenotypic description within this correlative study. Due to the limited amounts of available clinical material a more detailed analysis of centrosome function within separase-active patient cells with regard to functionality and genesis (unscheduled centrosome duplication vs. centrosome accumulation by failed cytokinesis) could not be performed and thus our correlative findings provide no basis for any functional statement.

On top of that, centrosomal clustering may also explain why aberrant centrosome numbers observed in MDS bone marrow cells may not necessarily lead to karyotype aberrations. Tumor cells have been reported to develop a centrosomal clustering mechanism to prevent multipolar spindle formation by coalescence of multiple centrosomes into two functional spindle poles [46–48]. Therefore, the aberrant amplification of even functional supernumerary centrosomes in MDS bone marrow cells may not instantly lead to aberrant karyotypes. Multiple rounds of cell divisions may be necessary to make the commencement of genetic instability via defective mitotic spindles evident.

Various conventional, hypomethylating or immunomodulating drugs are currently available for the treatment of MDS [31]. Two patients (#15, #36) of our follow-up cohort showed decreased SAD values under treatment with azacitidine. A similar effect was found in vitro, when MDS-L cells were treated with rigosertib, a strong inhibitor of Plk1 that induces G2/M arrest and plays a role in centriole disengagement by regulating separase activity at the level of substrate affinity at centrosomes [34, 49, 50]. The observation that elevated sAML-related SAD values can be “normalized” by anti-proliferative treatment with rigosertib correlating with the disappearance of the small cell population with prominent levels of separase proteolytic activity makes the SAD value a conceivable new marker for disease monitoring in MDS. However, while azacitidine treatment gave an effect in MDS patients no SAD value changes were observed in MDS-L cells after 48 h of azacitidine incubation. This may be due to the shorter incubation time of MDS-L cells when compared to the patient treatment schedule (48h vs. 7d) or to unknown missing factors that cannot be emulated in cell culture.

In summary, we found that the transformation from MDS to sAML coincides with the appearance of a small proportion of bone marrow cells displaying prominent levels (= high SAD value) of intracellular separase proteolytic activity. Hematopoietic cells derived from such patients exhibited an increased incidence of supernumerary centrosomes and irrespectively to the early stage of disease aberrant karyotypes were found in 27% of the specimen. We assume that increased separase proteolytic activity is due to failure in separase regulation and may be functionally associated with the capability of the leukemic clone to clonal evolution and disease progression. Common therapeutics can normalize high SAD values to levels found in MDS and healthy controls. Therefore, measurement of separase activity may be useful as surrogate marker of rampant proliferation for prediction or monitoring of the MDS transformation process. Our future studies will focus on the nature, time point of emergence, and the pathobiological role of isolated separase-overactive cells throughout disease progression from MDS to sAML. Moreover, the potential use of separase activity as a predictive marker needs to be validated in longitudinal studies.
